# Prescribing Smartphone Apps for Physical Activity Promotion in Primary Care: Modeling Study of Health Gain and Cost Savings

**DOI:** 10.2196/31702

**Published:** 2021-12-20

**Authors:** Leah Grout, Kendra Telfer, Nick Wilson, Christine Cleghorn, Anja Mizdrak

**Affiliations:** 1 Burden of Disease Epidemiology, Equity and Cost-Effectiveness Program University of Otago Wellington Wellington New Zealand

**Keywords:** physical activity, smartphone apps, mobile health, mHealth, modeling, primary care, mobile phone

## Abstract

**Background:**

Inadequate physical activity is a substantial cause of health loss worldwide, and this loss is attributable to diseases such as coronary heart disease, diabetes, stroke, and certain forms of cancer.

**Objective:**

This study aims to assess the potential impact of the prescription of smartphone apps in primary care settings on physical activity levels, health gains (in quality-adjusted life years [QALYs]), and health system costs in New Zealand (NZ).

**Methods:**

A proportional multistate lifetable model was used to estimate the change in physical activity levels and predict the resultant health gains in QALYs and health system costs over the remaining life span of the NZ population alive in 2011 at a 3% discount rate.

**Results:**

The modeled intervention resulted in an estimated 430 QALYs gained (95% uncertainty interval 320-550), with net cost savings of 2011 NZ $2.2 million (2011 US $1.5 million) over the remaining life span of the 2011 NZ population. On a per capita basis, QALY gains were generally larger in women than in men and larger in Māori than in non-Māori. The health impact and cost-effectiveness of the intervention were highly sensitive to assumptions on intervention uptake and decay. For example, the scenario analysis with the largest benefits, which assumed a 5-year maintenance of additional physical activity levels, delivered 1750 QALYs and 2011 NZ $22.5 million (2011 US $15.1 million) in cost savings.

**Conclusions:**

The prescription of smartphone apps for promoting physical activity in primary care settings is likely to generate modest health gains and cost savings at the population level in this high-income country. Such gains may increase with ongoing improvements in app design and increased health worker promotion of the apps to patients.

## Introduction

Inadequate physical activity is a risk factor for coronary heart disease (CHD), diabetes, stroke, and certain forms of cancer [1,2]. The World Health Organization recommends that adults aged 18 to 64 years should complete at least 150 minutes of moderate-intensity aerobic physical activity, at least 75 minutes of vigorous-intensity aerobic physical activity, or an equivalent combination of moderate- and vigorous-intensity aerobic physical activity each week [3]. Approximately 25% of adults do not meet the recommended level of physical activity worldwide, and it has been estimated that as many as 5 million deaths could be averted each year if the global population were more active [3].

In New Zealand (NZ), >40% of adults are estimated to be insufficiently physically active [4]. CHD, stroke, and diabetes are among the leading causes of health loss in NZ [5], and noncommunicable diseases contribute to marked health inequalities, with Māori (Indigenous population), Pasifika, and low-income New Zealanders at higher risk for important health conditions [5,6]. Strategies to increase physical activity at the population level are needed to help address this public health concern and reduce health inequalities.

In recent years, the use of mobile health (mHealth) tools to increase physical activity has risen [7,8]. Furthermore, the widespread use of mobile phones has made mHealth interventions scalable to a broad population [9,10]. Although there are a number of different mHealth tools and services available, smartphone apps may be a particularly popular approach to increasing physical activity.

In 2017, there were >325,000 health apps available from major app stores and approximately 3.7 billion app downloads worldwide [11]. The most popular health apps tend to be for diet, physical activity tracking, weight management, and adherence to medication [7,12,13]. Smartphone apps are generally considered easy to use and can enhance physical activity interventions through technological features (eg, accelerometers) [9]. Moreover, apps have been shown to be effective at increasing physical activity levels [10,14], although there is substantial variability in quality and effectiveness between the many available apps [15,16]. Physical activity apps also tend to be inexpensive or free of charge [10]. For example, in NZ, the Ministry of Health–supported web-based Health Navigator app library contains a number of different mHealth apps and specifically includes links to free and low-cost physical activity apps [17].

The prescription of physical activity apps during a primary care visit is a plausible intervention in the NZ context, as some general practitioners (GPs) already *prescribe* exercise as part of a green prescription program [18], although it is unclear whether there is substantial uptake of the program, and there are no requirements for physical activity levels to be assessed as part of standard care by GPs. Such a program could theoretically include smartphone app prescriptions. Clinicians and GPs already frequently recommend apps and other web-based resources during consultations [19,20], and the Royal New Zealand College of General Practitioners supports the adoption of such technology [21]. In addition, some NZ GPs recommend pedometer use for certain patients [22] and would presumably recommend the mHealth equivalent.

Given this background, this study assesses the health impacts, health system costs, and cost-effectiveness of the prescription of smartphone apps for the promotion of physical activity in primary care settings in NZ, a high-income country.

## Methods

### Modeling Methods

An established proportional multistate life table (PMSLT) model was used to estimate the health impact and health system expenditure of the prescription of smartphone apps for the promotion of physical activity in primary care [23,24]. Physical activity was measured as the change in the metabolic equivalent of task (MET) minutes per week of moderate and vigorous activity. The PMSLT model simulates the entire NZ population alive in 2011 (N=4.4 million) until death or the age of 110 years. Future all-cause morbidity and mortality and incidence and case fatality rates for 5 diseases related to changes in physical activity were projected. Specifically, the model included breast cancer (women only), colorectal cancer, CHD, type 2 diabetes, and stroke.

Health gain was measured in quality-adjusted life years (QALYs) [25], whereas, for costs, a health system perspective was used, and the outputs were the difference in total health system costs between business-as-usual and the modeled intervention and included the cost of implementing the intervention. We also disaggregated the results by period and presented the impact of the intervention after 10 years and 20 years. Calibration and validation of the epidemiological aspects of the PMSLT are described in a web-based technical report [23]. A Monte Carlo simulation (2000 iterations) was used to estimate the uncertainty intervals for the key results.

QALYs and costs were discounted at 3%, with results for 0% and 6% discount rates presented as scenario analyses. We also ran the results applying an *equity adjustment* that set background all-cause morbidity and mortality rates for Māori to non-Māori values [26]. This technique is often used to avoid the undervaluation of health gains and identify potential health equity impacts for Māori. Scenario analyses included higher percentages of the eligible population being screened for the intervention (ie, 25% and 50%), a reversed ratio of GP to practice nurse (PN) consultation time, and maintenance of the intervention impact for 5 years.

Full details of the model are published elsewhere [23,24,27].

### Intervention Specification

Rapid reviews of the literature on physical activity apps and referral schemes (ie, green prescription programs) were conducted to parameterize the intervention for modeling purposes. The modeling parameters used and the justification for their use are presented below (Tables 1 and 2).

**Figure 1:**
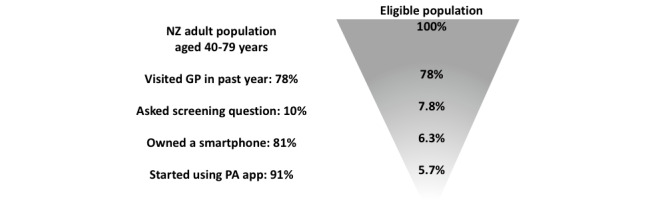
Flowchart of base-case intervention conceptualization for prescribed smartphone apps for physical activity promotion in primary care. GP: general practitioner; NZ: New Zealand; PA: physical activity.

As shown in Figure 1, during a GP visit, people with insufficient physical activity were identified using a screening question as per the one used by the NZ Health Survey [28]. For practical reasons and because it might be inappropriate to ask on some occasions, the screening question was assumed to only be asked during a proportion of such visits (10%, with 25% and 50% presented as scenario analyses). For those with insufficient levels of physical activity (defined as <300 MET minutes per week), the GP or PN then provided a printed physical activity prescription form that included specific instructions to exercise and information on how to download and use a physical activity smartphone app. The patient then chose a physical activity app, possibly with input or recommendations from the provider, from the Health Navigator app library. The Health Navigator app library is a repository of apps reviewed by experts, which contains links to a number of independently reviewed exercise apps [17]. It was assumed that individuals would use an app that was already developed and free to download. Furthermore, it was assumed that the app would be available at zero cost for as long as the participants chose to use it. However, adherence to the intervention was expected to decrease relatively quickly; therefore, long-term availability of the app would not be relevant for most participants. In addition to the prescription, GPs or PNs referred patients to service providers (eg, a regional sports trust or primary health organization in the NZ context) who provided support over the phone, including a comprehensive consultation, 2 brief follow-up calls, and technical support to use the app within the first year after the initial consultation and prescription.

We included direct costs attributable to the intervention (ie, intervention costs) and indirect costs attributable to changes in health system use resulting from the intervention [29]. Intervention costs were applied to everyone who received an initial consultation, even if they did not start using a physical activity app. In addition, it was assumed that all costs would be incurred in the first year following the initial consultation (ie, follow-up calls and technical support would occur in the same year as the intervention). Furthermore, it was assumed that those adults who were sufficiently active or who did not own a smartphone would require less than a minute of the GP’s time to screen; therefore, costs associated with such interactions were minimal and not quantified.

Current evidence indicates that physical activity interventions would likely provide minimal chronic disease reduction benefits to adults aged <40 years in NZ [24,27]. Therefore, the age range for the intervention was restricted to 40 to 79 years. Individuals aged ≥80 years were excluded because of low smartphone ownership and use, as well as the relatively high prevalence of comorbidities (eg, arthritis) that could limit participation in physical activity. Although the intervention was only applied to those aged 40 to 79 years, the model followed the entire NZ population alive in 2011 over their remaining life span. Therefore, participants could age into the intervention over time (eg, someone who was aged 38 years in 2011 could still become eligible for the intervention in 2013 when they turned 40).

On the basis of evidence in the literature (Table 1), it was assumed that 91% of eligible patients who were active smartphone users would start the intervention and that physical activity would increase by an average of 410 moderate to vigorous physical activity MET minutes per week. After 1 year, the intervention effect was assumed to be maintained by 36.5% of people, with a decay rate of 55% applied.

### Parameters

Tables 1 and 2 contain additional details on the base-case intervention parameters.

**Table 1:** Modeling input parameters for the prescription of smartphone apps for physical activity promotion in primary care.

Parameter and key source			Supporting evidence and notes		Value (UI^a^; beta distribution unless otherwise indicated)		Resulting percentage (alternate scenarios)
**Visited GP^b^ in the past year**							
	Ministry of Health [30]	According to the NZ^c^ Health Survey (NZHS), 78% of NZ adults (aged ≥15 years) visited their GP in the past year [30]. The uncertainty intervals are an assumed percentage (±10%).		78% (68%-88%)		78%	
**Asked screening question**							
	Croteau et al [31]	A national survey on physical activity and nutrition in NZ by Croteau et al [31] found that only 3% of the survey population (n=235) reported receiving a green prescription from a GP or PN^d^. In addition, the study reported that those aged ≥45 years were significantly more likely to have received a green prescription. On the basis of the findings from Croteau et al [31] and survey data from the NZHS, which indicated that 49% of people who visit their GP have insufficient levels of physical activity [30], it was back-calculated that only approximately 10% of the eligible population would be asked the physical activity screening question during any of their GP visits within a year (ie, the estimate for NZ adults who visited their GP in the previous year [78%] multiplied by the estimate for those asked the screening question [10%], the estimate by Croteau et al [31] for insufficient physical activity [49%], and uptake of the app [81%] equals 3%, which is the percentage of the eligible population that would be expected to receive a green prescription from a GP or PN). However, several alternate scenarios with higher percentages of the eligible population being asked the screening question are also considered in scenario analysis. Although no other evidence was found to support parameter selection, incomplete screening was still included for several reasons. For example, the patient may not be able to exercise because of an existing health condition. In addition, it may be inappropriate to ask the screening question during a consultation about an urgent and critical other matter, and the GP may not have the time for noncritical care provision.		10% per year (alternate scenario 1: 25%; alternate scenario 2: 50%)		7.8% of the eligible population (alternate scenario 1: 19.5%; alternate scenario 2: 39%)	
**Smartphone ownership**							
	DataReportal [32]	Using recent metrics on NZ smartphone ownership (based on Google Consumer Barometer data), it has been reported that 81% of NZ adults own a smartphone [32]. The uncertainty intervals are an assumed percentage (±5% of the point estimate).		81% (77%-85.1%)		6.3% of the eligible population (alternate scenario 1: 15.8%; alternate scenario 2: 31.6%)	
**Uptake of the smartphone app**							
	Glynn et al [33]	On the basis of an RCT^e^ of physical activity apps prescribed in primary care in Ireland, 91% of eligible patients who were active smartphone users were assumed to start the intervention [33]. The uncertainty intervals are an assumed percentage (±10% of the point estimate).		91% (81.9%-100%)		5.7% of the eligible population (alternate scenario 1: 14.4%; alternate scenario 2: 28.8%)	
**Increase in physical activity**							
	Glynn et al [33]	On average, mHealth^f^ physical activity interventions result in an increase in physical activity, at least in the short term [10,14,34]. As a result of the intervention, it was assumed that physical activity would increase on average by 410 MVPA^g^ MET^h^ minutes per week. This total was taken from an RCT studying a GP-prescribed physical activity app and follow-up support [33]. The study reported a 2017-step increase per day after accounting for differences between the intervention and control groups. Steps per day were converted to MVPA MET minutes per week using the method outlined in a web-based report [35]. The *Steps to MVPA Conversion* section of this report also has further details about the formula used. The uncertainty intervals are an assumed percentage (±10% of the point estimate).		Increase by 410 (369-451) minutes of MVPA MET minutes per week; normal distribution		—^i^	
**Adherence at 1 year**							
	Allman-Farinelli et al [36] and Damschroder et al [37]	After 1 year, the intervention effect was assumed to be maintained by 36.5% of people. This was based on the average of estimates for 2 app+ (ie, a smartphone app in addition to follow-up texts, calls, or emails) intervention studies (see below), which typically fall between the estimates for traditional green prescription programs and app-only physical activity interventions in retention and adherence. The uncertainty intervals are an assumed percentage (±20% of the point estimate). The first RCT for the prevention of weight gain in young adults in Australia found that an app+ intervention that targeted both dietary behaviors and physical activity generated a 40% response rate to follow-up SMS text messages at 9 months [36]. The second RCT for physical activity in US veterans reported a 33% retention rate at 12 months for an app+ group that received follow-up phone calls [37].		36.5% (29.2%-43.8%)		—	
**Decay rate of intervention effect after 1 year**							
	Gc et al [38]	A recent modeling study of brief physical activity interventions also used a similar methodological approach and assumed that the interventions had an effect for the first year and then applied a 55% decay rate every year afterward [38]. This was in line with several previously reported physical activity modeling studies (ie, Over et al [39], Cobiac et al [40], and Jacobs-van der Bruggen et al [41]) that assumed similar base-case decay rates, varying between 50% and 55%. The uncertainty intervals are an assumed percentage (±20%).		55% (35%-75%)		—	

^a^UI: uncertainty interval.

^b^GP: general practitioner.

^c^NZ: New Zealand.

^d^PN: practice nurse.

^e^RCT: randomized controlled trial.

^f^mHealth: mobile health.

^g^MVPA: moderate to vigorous physical activity.

^h^MET: metabolic equivalent of task.

^i^Not available (does not change % of eligible population).

**Table 2:** Cost input parameters for the prescription of smartphone apps for physical activity promotion in primary care.

Parameter			Key source		Supporting evidence and notes		Value (95% UI^a^)
Ratio of GP^b^ to PN^c^ consultations			Research New Zealand [42]		Approximately 73% of consultations were assumed to be GP-run and the rest were run by PNs. These proportions are based on the referral sources reported by the NZ^d^ Green Prescription Patient Survey [42].		73% GP, 27% PN; however, in a scenario analysis, this ratio was reversed.
**GP consultation parameters**							
	GP consultation time	Elley et al [43]		On the basis of an RCT^e^ studying the NZ Green Prescription Program [43], 7 minutes of GP time were spent on the physical activity advice and prescription part of each consultation. Although it is likely that the overall consultation will typically be longer, only the physical activity–specific part has been quantified. Other studies have reported a longer duration [38]; however, NZ-specific data were used for this parameter. It was assumed that there would be additional GP time available for the intervention and, therefore, all other patient concerns would still be covered in the appointment, and no adverse effects would arise from the GP consultation. The uncertainty intervals are an assumed percentage (±10% of the point estimate).		7 minutes (6.3-7.7)	
	Cost of GP consultation in 2011	Association of Salaried Medical Specialists [44]		The cost of 7 minutes of a GP consultation was assumed to be NZ $15.38 (US $10.35), or NZ $2.20 (US $1.48) per minute. The midpoint of a GP annual salary scale in 2018 was taken from the Wellington Union Health Services Collective Agreement [44]. An hourly rate of NZ $94.15 was then calculated using this estimate. With 50% overheads, this equates to NZ $141.20, or NZ $131.85 in 2011 adjusted for inflation [45]. For 7 minutes of a consultation at an hourly rate of NZ $131.85, the physical activity part of the GP consultation would cost NZ $15.38 per consultation. By comparison, the NZ government agency PHARMAC estimated that the cost of a GP practice visit was NZ $80 per consultation in 2018 [46]. This equates to NZ $73.73 per consultation in 2011 [45], and NZ $33.73 once a patient copayment of NZ $40 is removed. If 7 out of 15 minutes were allocated to physical activity advice and prescription, the cost would be approximately NZ $15.74, which is close to the estimate above. The final costs have been presented as 2011 NZ $. The baseline year of the model was 2011, and cost parameters were consumer price index–adjusted to the 2011 NZ $ to reflect this. With the exception of costs, other parameters in this table are more current, so they produce more relevant outputs.		NZ $2.20/minute (US $1.48/minute)	
**PN consultation parameters**							
	PN consultation time	Elley et al [43]		A PN was assumed to spend approximately 13 minutes on the physical activity app consultation based on the results of an RCT on the NZ Green Prescription Program [43]. It was assumed that there would be additional PN time available for the intervention and, therefore, all other patient concerns would still be covered in the appointment, and no adverse effects would arise from the consultation. The uncertainty intervals are an assumed percentage (±10% of the point estimate).		13 minutes (11.7-14.3)	
	Cost of PN consultation in 2011	Elley et al [47]		The cost of a 13-minute consultation was assumed to be NZ $8.27 (2011 US $5.57), consumer price index–adjusted to the 2011 NZ $, or NZ $0.64 (US $0.43) per minute. A PN hourly wage was NZ $19.12/hour in 2000-2001 [47], equivalent to NZ $25.42 in 2011 [45]. This equates to NZ $38.16 per hour with overheads (as per the GP calculations) and NZ $8.27 for a 13-minute consultation. By comparison, the midpoint of the Practice Nurse Collective Employment Agreement pay scale was NZ $24.36 per hour [48]. This is similar to our hourly rate before adjusting for overheads.		NZ $0.64/minute (US $0.43/minute)	
**Additional costs^f^**							
	Cost of additional resources in 2011	Elley et al [47]		The cost of follow-up phone calls and additional resources was assumed to be NZ $90.10 (US $60.63) per individual. As per the structure of the NZ Green Prescription Program, the intervention was assumed to also include phone calls and additional resource use. After consultation, the intervention would include 3 follow-up phone calls, the first a comprehensive consultation and then 2 brief follow-up calls. The phone calls would include general advice on physical activity and technical support to use the app. Additional resources would include educational material dissemination, such as an email with a link to a website with responses to frequently asked questions. Similar services were estimated to cost NZ $69 per person in 2001-2002 based on the results of a PhD thesis on the NZ Green Prescription Program [47]. This NZ $69 in 2001 equates to NZ $90.10 (US $60.63) in 2011 [45]. The uncertainty intervals are an assumed percentage (±10% of the point estimate).		NZ $90.10 (US $60.63; 81.09-99.11)	

^a^UI: uncertainty interval.

^b^GP: general practitioner.

^c^PN: practice nurse.

^d^NZ: New Zealand.

^e^RCT: randomized controlled trial.

^f^It was assumed that individuals would use an app that was already developed and was free to download from the Health Navigator website (ie, zero cost for the app); it was also assumed that there was zero cost for promoting the app to primary care workers.

## Results

The prescription of smartphone apps for physical activity promotion in primary care resulted in an increase of 430 QALYs (95% uncertainty interval 320-550) over the lifetime of the 2011 NZ population. This was equivalent to 0.13 QALYs gained per 1000 population (95% uncertainty interval 0.01-0.16; Table 3) or 0.23 QALYs gained per 1000 adults aged 40 to 79 years. Of the total, 150 QALYs and health system cost savings of NZ $174,000 (US $117,000) were accumulated in the first 10 years following intervention implementation, and 160 QALYs and cost savings of NZ $1,961,000 (US $1,320,000) were accumulated in the 20 years following implementation.

The modeled improvements in health came with net cost savings of NZ $2.2 million (US $1.5 million). The intervention was cost-saving for all age-sex-ethnicity subgroups, except for non-Māori women aged 40 to 59 years.

On a per capita basis, QALY gains were generally larger in women than in men, larger in Māori than in non-Māori, and largest in the 60 to 79 years age group. Health gains for Māori increased with the application of the equity adjustment (ie, non-Māori morbidity and mortality rates used for Māori; Table 4).

In the first 10 years after the intervention was implemented (2011-2020), the total health gain was 148 QALYs, with net cost savings of NZ $174,000 (US $117,000) for the health system. After 20 years (2011-2030), the total health gain was 158 QALYs, with net health system cost savings of NZ $1.96 million (US $1.32 million).

The impact of selected changes to model specifications on the results was explored (Table 5). Changing the discount rate had the expected impact on the overall results, with a 6% discount resulting in smaller health gains and cost savings and a 0% discount (ie, undiscounted) resulting in larger health gains and cost savings. Increasing the percentage of primary care patients who were asked the screening question for the intervention also had the expected impact on the overall results, with higher screening rates resulting in higher health gains. However, the cost savings were larger for the scenario in which 25% of patients were asked the screening question (ie, cost savings of NZ $3.3 million [US $2.2 million]) than for the scenario in which 50% were asked (ie, cost savings of NZ $2.6 million [US $1.8 million]) because of the cost of intervention implementation. Dominant provision by PNs (reversing the ratio of GP to PN consultation provision) resulted in a small increase in cost savings (NZ $148,000 [US $100,000]) over the base case scenario. Assuming that the intervention impact would be maintained for 5 years following the intervention (rather than for 1 year), the health gains were estimated to be >4 times higher than in the base-case analysis and would result in much higher cost savings (NZ $22.5 million [US $15.1 million]).

**Figure 2:**
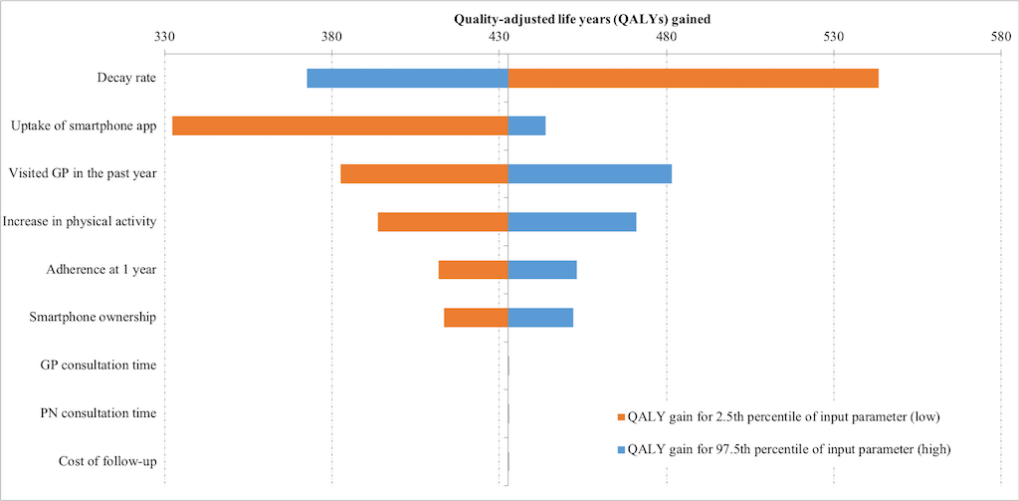
Tornado plot showing the contribution of parameter uncertainty to overall uncertainty in the quality-adjusted life years gained for the studied adult population. GP: general practitioner; PN: practice nurse; QALY: quality-adjusted life year.

**Figure 3:**
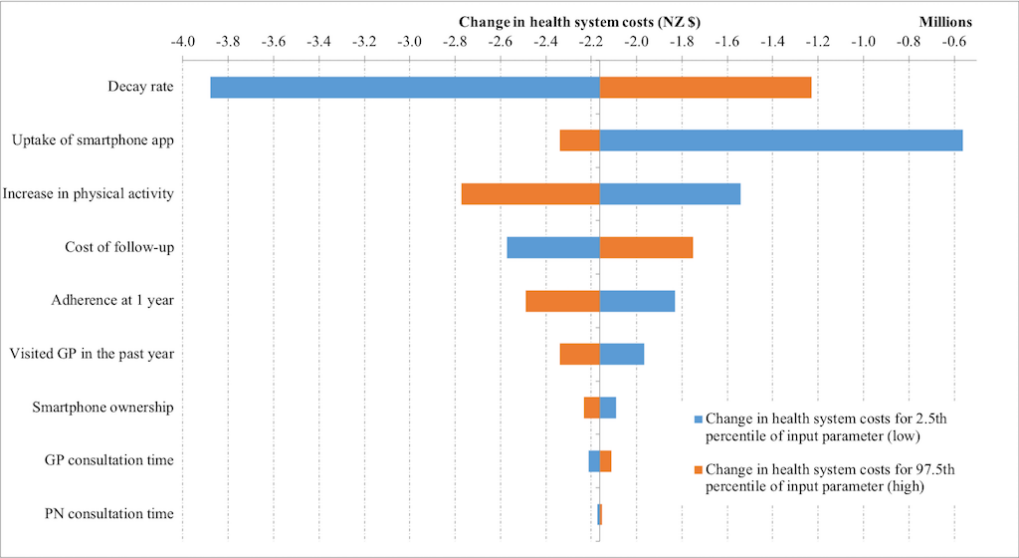
Tornado plot showing the contribution of parameter uncertainty to overall uncertainty in the change in health system costs. GP: general practitioner; PN: practice nurse. 2011 NZ $1=2011 US $0.67.

Finally, we examined the contribution of individual intervention parameters to the uncertainty in the modeled results. Uncertainty in health gains and health system cost impacts was primarily driven by the decay rate and the uptake of the smartphone app (Figures 2 and 3).

**Table 3:** Health gains and health system costs of the prescription of smartphone apps for physical activity promotion in primary care by age, sex, and ethnicity (lifetime gains and 3% discount rate). 2011 NZ $1=2011 US $0.67.

Sex, ethnicity, and age group				Health gain, QALYs^a^ (95% UI^b^)		QALYs/1000 population (95% UI)		Health system costs, 2011 NZ $ million/2011 US $ million (95% UI)
**All sexes and all ethnicities**								
	All age groups			430 (320 to 550)		0.13 (0.10 to 0.16)		−2.16^c^/−1.45 (−4.49 to −0.11)
	40-79 years			430		0.23		−2.16 /−1.45
**Male**								
	**Non-** **Māori**							
		40-59 years	69 (50 to 89)		0.13 (0.10 to 0.17)		−0.34/−0.23 (−0.83 to 0.09)	
		60-79 years	103 (75 to 140)		0.34 (0.25 to 0.45)		−0.79/−0.53 (−1.38 to −0.28)	
	**Māori**							
		40-59 years	19 (14 to 25)		0.30 (0.22 to 0.39)		−0.17/−0.11 (−0.27 to −0.08)	
		60-79 years	9 (7 to 12)		0.42 (0.31 to 0.55)		−0.09/−0.06 (−0.14 to −0.05)	
**Female**								
	**Non-Māori**							
		40-59 years	58 (43 to 76)		0.11 (0.08 to 0.14)		0.17/0.12 (−0.28 to 0.60)	
		60-79 years	130 (96 to 170)		0.41 (0.30 to 0.54)		−0.69/−0.46 (−1.30 to −0.13)	
	**Māori**							
		40-59 years	24 (17 to 31)		0.33 (0.24 to 0.42)		−0.18/−0.12 (−0.30 to −0.06)	
		60-79 years	14 (10 to 18)		0.56 (0.41 to 0.73)		−0.08/−0.05 (−0.14 to −0.03)	

^a^QALY: quality-adjusted life year.

^b^UI: uncertainty interval.

^c^Negative cost (ie, the intervention results in cost savings to the health system).

**Table 4:** Results for Māori (Indigenous population) with equity adjustment applied (40-79 age group, lifetime gains, and 3% discount rate).

Sex and age group		Health gain, QALYs^a^ (95% UI^b^)	QALYs/1000 population (95% UI)	Health system costs, NZ $ million/US $ million (95% UI)
**Male**				
	40-59 years	24 (18 to 31)	0.37 (0.28 to 0.49)	−0.17/−0.11 (−0.28 to −0.09)
	60-79 years	13 (10 to 17)	0.59 (0.44 to 0.77)	−0.09/−0.06 (−0.15 to −0.05)
**Female**				
	40-59 years	29 (22 to 38)	0.40 (0.30 to 0.51)	−0.18/−0.12 (−0.32 to −0.08)
	60-79 years	20 (14 to 25)	0.78 (0.58 to 1.01)	−0.08/−0.06 (−0.15 to −0.03)

^a^QALY: quality-adjusted life year.

^b^UI: uncertainty interval.

**Table 5:** Sensitivity and scenario analyses for the prescription of smartphone apps for physical activity promotion in primary care (expected value analysis, lifetime perspective, and 3% discount rate unless otherwise noted).

Sensitivity and scenario analyses	Health gains, QALYs^a^	Net health system costs, NZ $ million (US $ million)	Cost per QALY gained, NZ $
Base-case analysis	430	−2.162 (−1.455)	Cost saving^b^
Undiscounted	720	−3.820 (−2.571)	Cost saving
6% discount rate	290	−0.900 (−0.605)	Cost saving
25% asked screening question	950	−3.339 (−2.247)	Cost saving
50% asked screening question	1640	−2.644 (−1.779)	Cost saving
Dominant provision by PNs^c^ (reversed ratio of GP^d^ to PN consultations)	430	−2.310 (−1.555)	Cost saving
5-year maintenance of additional physical activity levels followed by a return to preintervention levels (otherwise base case)	1750	−22.490 (−15.135)	Cost saving

^a^QALY: quality-adjusted life year.

^b^Negative cost per QALY gained (ie, the intervention results in cost savings to the health system).

^c^PN: practice nurse.

^d^GP: general practitioner.

## Discussion

### Principal Findings and Interpretation

The potential impact of the prescription of smartphone apps for physical activity promotion in primary care settings in NZ was modeled using published estimates of uptake, effectiveness, and adherence [33,36,37]. The total health impact was modest, with 430 QALYs gained over the remaining life span of the population, albeit up to 1750 QALYs if the intervention effect was maintained for 5 years. The intervention was also likely to be cost saving for the health system and provide larger per capita health gains for Māori than for non-Māori. Given the higher per capita health gains for Māori than for non-Māori, it is plausible that this intervention could play a role in reducing health inequalities if implemented equitably. Although there is strong evidence that physical activity levels can be improved and maintained in the short term through individual-level physical activity interventions, a cumulative meta-analysis of the effects of physical activity interventions found that additional research is needed to identify interventions that are the most cost-effective [49]. This study provides additional modeling evidence of the cost-effectiveness of physical activity promotion in a primary care setting.

### Comparison With Prior Work

When compared with selected health interventions in NZ, according to methodologically compatible modeling studies, we found that the prescription of smartphone apps for physical activity promotion in primary care was likely to provide larger health gains and cost savings for the health system than a mass media campaign for physical activity apps [27], a mass media campaign for weight loss apps [50], or weight loss counseling by nurses in primary care [51] in NZ (Table 6). However, the intervention was less effective than a mass media campaign to promote a smoking cessation app in NZ [52]. It was also less effective on a per capita basis than a traditional *green prescription* program to promote physical activity in Australia [40], although this may be due in part to underlying differences in physical activity patterns and epidemiology across different populations. We also note that the health gains of these individual-level interventions are orders of magnitude lower than upstream interventions (eg, tobacco control endgame interventions [53] and switching driving trips to walking and cycling [24]). Implementing the prescription of smartphone apps for physical activity promotion in primary care alongside other such interventions may help maximize health gains.

**Table 6:** Comparison of the impact of various health interventions in New Zealand according to methodologically compatible epidemiological and health economic modeling (lifetime perspective and 3% discount rate).

Intervention	Health gains, QALYs^a^	Net health system costs, NZ $ million^b^	Cost per QALY gained (incremental cost-effectiveness ratio), NZ $^b^
Prescription of smartphone apps for physical activity promotion in primary care (this study)	430	−2.2	Cost saving
Mass media campaign to promote physical activity apps [27]	28	2.2	81,000
Mass media campaign to promote weight loss apps [50]	29	2.9	79,700
Weight loss dietary counseling by nurses in primary care [51]	250	38.8	138,000
5-year mass media campaign to promote smoking cessation app [52]	6760	−115.0	Cost saving
Enhanced green prescription program among women aged 40-74 years [54]	—^c^	—^c^	687^d^

^a^QALY: quality-adjusted life year.

^b^2011 NZ $1=2011 US $0.67.

^c^This study did not use the same modeling approach but calculated cost-effectiveness ratios.

^d^Program cost per person made *active* and sustained at 12 months.

### Study Strengths and Limitations

A strength of this study was the use of an established PMSLT model for physical activity [24,27], which is based on high-quality disease-specific epidemiological and costing data for a whole country. The modeling framework has been widely used in Australia and NZ to assess different health interventions, including several different mHealth interventions [27,50,52], allowing for comparisons across studies. However, a limitation of the PMSLT approach is the assumption of disease independence, although the model does account for the relationship between type 2 diabetes and CHD and stroke, given that type 2 diabetes is a risk factor for these conditions. The model also does not account for potentially beneficial health impacts via the reduction of obesity, anxiety, or depression (or, conversely, health loss via an increase in these conditions because of musculoskeletal injuries associated with physical activity). Another limitation of this study was the use of a health system perspective for costs and benefits. Such a perspective does not allow for the estimation of societal-level costs and benefits outside of the health system, such as impacts on household spending or reduced greenhouse gas emissions from transport mode shifts.

There are also limitations associated with the parameter estimates used. For example, app uptake likely varies and could be increased with improved app design. In addition, adherence estimates in the literature range widely. We examined estimates from green prescription programs, stand-alone app interventions, and app+ interventions (ie, a smartphone app in addition to follow-up texts, calls, or emails). We chose an adherence estimate between those for green prescription programs and app-only interventions, which was in line with app+ interventions in which there would be some level of follow-up with the intervention participants. We assumed that all of the intervention parameters presented in Tables 1 and 2, including intervention uptake and adherence, would be the same across population groups (ie, men and women and Māori and non-Māori). However, it is possible that for certain parameters, there may be significant variations across population groups that may impact the effectiveness of the intervention. For example, it is unclear whether currently available apps adequately cater to the needs of diverse population groups.

### Potential Implications for Research

Additional research is needed to optimize interventions that can lead to sustained increases in physical activity levels over the long term [48] and identify the forms of follow-up (eg, phone calls vs emails) that maximize adherence to physical activity interventions. App improvements may also encourage adherence. Evidence suggests that apps are most effective when they incorporate self-monitoring of physical activity, reminders for app use, and social interaction with peers [7,55]. Gamification (eg, providing point-based rewards for frequency or consistency of app use) may also improve outcomes for app-based interventions [56]. Other factors that may influence app use include simple app interfaces, easy navigation, and automatic or simplified data entry [57]. Such features help increase user engagement, which promotes positive behaviors [7]. Advancements in app design merit additional research to assess their impact on adherence.

### Potential Implications for Policy

There are indications that such a program would be best administered by PNs rather than GPs, given the larger cost savings associated with the scenario in which PNs are the dominant deliverers of the consultation. In addition, the literature suggests that GPs are often particularly time-limited [58]. Our scenario analyses suggest that larger health gains would be achievable if a higher percentage of patients were asked the screening question (ie, 25% or 50% asked the screening question). If GPs are unable to ask the screening question as frequently as PNs because of time constraints, then it may be both more effective and a better use of staff resources to routinely have PNs deliver such a program. This may also have implications for the administration of the ongoing green prescription program in NZ. However, the ratio of GP to PN consultations was only included in the model as a cost parameter, and we did not assess potential differences in effectiveness between GP and PN administration of the program. We also note that, in the context of high levels of unmet need for health care (eg, 13.3% of NZ adults did not visit a GP because of cost barriers in 2019-2020 [59]) and differential access to smartphones and the internet, additional strategies to ensure that any app-based intervention does not exacerbate existing health inequities are warranted.

With the widespread use of smartphones, mHealth interventions such as this have a large potential for scalability to a broad population [9,10]. As part of a range of interventions to address insufficient physical activity, governments should consider investing in the promotion of physical activity smartphone apps, along with additional research to improve app effectiveness and uptake. The intervention should ideally be tailored to the country context, which may include examining existing structural inequities that may influence intervention success and co-designing strategies with relevant population groups.

The promotion of smartphone apps may also complement other strategies to promote physical activity (eg, investments in walking and cycling infrastructure [60]) to support long-term behavioral changes. Although our results suggest that the promotion of physical activity smartphone apps in primary care is likely to be effective and cost-saving in NZ, these results are also likely generalizable to other high-income countries with similar chronic disease epidemiology, physical activity levels, and other population characteristics.

### Conclusions

In this modeling study, the prescription of smartphone apps for physical activity promotion in primary care in NZ yielded modest health gains and was cost saving for the health care system. The scope for this type of mHealth intervention is expanding with the increase in smartphone ownership and the availability of easy-to-use and effective apps. This intervention should be considered by policy makers in NZ and also be considered by other high-income nations with similar characteristics.

## Abbreviations

CHD: coronary heart disease
GP: general practitioner
MET: metabolic equivalent of task
mHealth: mobile health
NZ: New Zealand
PMSLT: proportional multistate life table
PN: practice nurse
QALY: quality-adjusted life year
